# Relationship between sleep quality and quantity and lower-body neuromuscular performance characteristics in semi-professional male basketball players

**DOI:** 10.3389/fspor.2024.1439858

**Published:** 2024-08-13

**Authors:** Damjana V. Cabarkapa, Dimitrije Cabarkapa, Andrew C. Fry

**Affiliations:** Jayhawk Athletic Performance Laboratory—Wu Tsai Human Performance Alliance, Department of Health, Sport and Exercise Sciences, University of Kansas, Lawrence, KS, United States

**Keywords:** force, power, PSQI, recovery, fatigue, training, eccentric, concentric

## Abstract

Sleep has been recognized as one of the most essential recovery methods necessary for achieving optimal performance. However, there is still a lack of scientific literature focused on examining its impact on one of the most prevalent skills in the game of basketball, the countermovement vertical jump (CVJ). Therefore, the purpose of the present study was to examine the relationship between sleep quality and quantity, and lower-body neuromuscular performance characteristics within a cohort of semi-professional male basketball players. Twenty-eight athletes competing in a first-tier regional league in Serbia volunteered to participate in this investigation. Upon arrival at the gym, all athletes completed the Pittsburgh Sleep Quality Index (PSQI) self-rated questionnaire. Immediately after completion of the PSQI, each athlete stepped on a force plate system and performed three maximum-effort CVJs with no arm swing. The following force-time metrics were obtained for the analysis: eccentric and concentric absolute and relative mean and peak force and power, vertical jump height, and reactive strength index-modified. Pearson product-moment correlation coefficients were used to examine the strength of the linear relationships between sleep quality and quantity and lower-body neuromuscular performance characteristics (*p *< 0.05). The results indicated that sleep quality appears to have a greater impact on the concentric than the eccentric phase of the CVJ (e.g., concentric mean force [r = −0.830; *p *< 0.001], relative concentric peak force [r = −0.466; *p *= 0.013, eccentric mean power (r = −0.162; *p *= 0.409)], while no significant relationship was found between sleep quantity and lower-body neuromuscular performance (e.g., concentric peak force [r = −0.055; *p *= 0.782], relative eccentric mean power [r = −0.301; *p *= 0.107]). Overall, these findings offer valuable insights into the importance of good sleep hygiene (e.g., efficiency, duration) in an athletic population, and can help practitioners develop more effective training and recovery programs.

## 1 Introduction

Basketball is one of the most popular team sports characterized by high-intensity actions such as repeated accelerations, decelerations, change of direction movements, and jumps (1–5). While the game of basketball places a significant amount of stress on athletes, it is important to consider other factors such as travel, sleep, and recovery when analyzing athletes’ fatigue levels and their overall physiological and psychological status (3, 6).

Sleep has been recognized as one of the most essential recovery methods that can significantly impact athletic performance (3, 7). Unlike the other recovery strategies that are often implemented within sports settings such as dietary supplements, compression garments, massage, and cold-water immersion (6), sleep has no cost and is accessible to everyone (8). Over recent years, various tools have been used by practitioners to monitor sleep such as polysomnography (PSG), actigraphy, commercial sleep technology, smartphone applications, sleep diaries, and sleep questionnaires (7). PSG has been often utilized in research settings for the assessment of sleep disorders and has been considered a gold standard for sleep analysis (7, 9). However, this method is quite expensive and complex to use (7). Thus, more user-friendly and cost-effective tools have been implemented in the applied settings by practitioners. One such example is the Pittsburgh Sleep Quality Index (PSQI) self-rated questionnaire that has been validated and established in the field and is frequently used to examine sleep quality over a one-month period (7, 9, 10).

Previous research has examined the impact of sleep duration and quality on athletes’ anaerobic (11, 12), aerobic (13), and cognitive performance (14) as well as on their mood and reaction time (8). Specifically, Mah et al. (8) examined how the increase in total sleep duration over multiple weeks impacted reaction time, mood, and daytime sleepiness within a cohort of recreationally active males playing on a varsity basketball team. It was observed that extended sleep time resulted in faster sprint times (∼1 s), better shooting accuracy (i.e., 9% increase in free throw and 9.2% increase in three-point shooting percentage), faster reaction times (∼0.3–0.5 s) and less daytime sleepiness when compared to the baseline scores (8). Similar findings were observed by Mougin et al. (13), where partial sleep deprivation significantly altered aerobic performance by increasing the heart rate, ventilation, and ventilatory equivalent to the VO_2_ ratio at submaximal (75% of VO_2_) and maximal workloads. On the other hand, Souissi et al. (12) showed that following 24 h of sleep deprivation, participants did not experience significant alterations in the Wingate and force-velocity tests. However, their anaerobic performance was impaired following the 36 h of sleep deprivation (12). Lastly, when examining the impact of late-night social media activity on the next-day game performance at the National Basketball Association (NBA) level of competition, Jones et al. (15) found that players who spent more time on social media during their evenings were more sleep deprived, which negatively impacted their basketball performance, ultimately resulting in fewer points and rebounds during the game. Thus, it can be implied that sleep plays a significant role in athletes’ recovery and is necessary in order to achieve optimal performance.

However, while the aforementioned investigations offer valuable insight into the impact of sleep on optimal performance, there is still a lack of scientific literature focused on examining its impact on one of the most prevalent skills and tests in the game of basketball, the countermovement vertical jump (CVJ). Hence, with force plate systems being widely used in practical settings for the assessment of CVJ and with their ability to provide practitioners with a comprehensive analysis of neuromuscular performance during both eccentric and concentric phases of the jumping movement, the purpose of the present study was to examine the relationship between sleep quality and quantity assessed via PSQI, and lower-body neuromuscular performance characteristics within a cohort of semi-professional male basketball players. It is hypothesized that sleep-related scores would demonstrate moderate to strong correlations with different CVJ force-time metrics.

## 2 Materials and methods

### 2.1 Participants

Twenty-eight semi-professional male basketball players competing in a first-tier regional league in Serbia volunteered to participate in the present investigation (age = 22.8 ± 3.7 years; body mass = 85.4 ± 10.6 kg; height = 189.1 ± 7.7 cm). The inclusion criteria involved all athletes free of musculoskeletal injuries who were cleared for participation in team activities by their respective sports medicine staff. If athletes did not meet the aforementioned criteria, they were excluded from participation. The testing procedures performed in this investigation were previously approved by the University of Kansas Institutional Review Board (No. 00149094) and all participants signed an informed consent document.

### 2.2 Procedures

The testing procedures were completed during the mid-season competitive period, 48–72 h following the last game played during the week when the performance assessment occurred (18:00–19:30 h). Upon arrival at the gym for regular team practice, athletes were thoroughly familiarized with the testing procedures, including PSQI guidelines and the CVJ testing protocol.

The PSQI questionnaire entailed 19 self-reported items belonging to one of the following categories: (a) subjective sleep quality, (b) sleep latency, (c) sleep duration, (d) habitual sleep efficiency, (e) sleep disturbances, (f) use of sleeping medication, and (g) daytime dysfunction (10). Likert-type questions were used to assess sleep quality and quantity over the time period of the past month. Following the PSQI-specific guidelines, scores for each question were transferred to a 0–3 severity scale, ultimately leading to a global score that could range between 0 and 21 (10). A score of 0 indicated optimal sleep pattern, while any increase in the score coincided with a decrease in sleep quality and/or quantity. In addition to the overall PSQI score, hours of sleep were included in the correlational analysis as a separate metric.

Following the completion of PSQI, all athletes completed a standardized warm-up procedure (10–15 min) administered by the team's respective strength and conditioning coach, consisting of dynamic stretching exercises (e.g., butt kicks, A-skips, high-knees) and basketball-specific-movements (e.g., low-to-moderate intensity jog and change-of-direction drills). Then, each athlete stepped on a uni-axial force plate system (ForceDecks Max, VALD Performance, Brisbane, Australia) sampling at 1,000 Hz and performed three maximum-effort CVJs with no arm swing (i.e., hands on the hips during the entire CVJ movement). Strong verbal encouragement was provided while the athletes were instructed to focus on pushing the ground as forcefully as possible (16). The force plate system was re-calibrated between each participant and the mean value across three jump trials was used for performance analysis purposes. The force-time metrics examined in the present study were eccentric and concentric absolute and relative mean and peak force and power, vertical jump height (i.e., impulse-momentum calculation), and reactive strength index (RSI)-modified (i.e., jump height divided by contraction time). The validity and reliability of the aforementioned metrics can be found in the previously published research reports (17–23) and a detailed explanation at https://valdperformance.com.

### 2.3 Statistical analysis

Means and standard deviations (x¯ ± SD), were calculated for each dependent variable for descriptive purposes. Shapiro-Wilk and Q-Q plots were used to examine if the assumption of normality was not violated. Pearson product-moment correlation coefficients (r) were used to examine the strength of the linear relationships between sleep quality and quantity and lower-body neuromuscular performance characteristics, separately for each dependent variable. The magnitude correlation thresholds were interpreted as follows: 0.0–0.1—trivial; 0.1–0.3—small; 0.3–0.5—moderate; 0.5–0.7—strong; 0.7–0.9—very strong; 0.9–1.0—near-perfect to perfect (24, 25). Statistical significance was set *a priori* to *p *< 0.05. All statistical analyses were completed with SPSS (Version 26.0; IBM Corp., Armonk, NY, USA).

## 3 Results

The PSQI score (x¯ ± SD; 5.0 ± 2.1) revealed a moderate-strong statistically significant negative correlation with relative and absolute concentric mean and peak force and power, absolute and relative eccentric mean force, absolute eccentric peak force, and RSI-modified. However, no significant relationship was observed between the PSQI score and the remaining eccentric variables examined in the present study (i.e., relative eccentric peak force, absolute and relative eccentric mean and peak power), as well as the vertical jump height. Also, no significant association has been detected between any of the aforementioned CVJ force-time metrics and the number of hours that athletes slept each night over a one-month period (7.9 ± 0.9). See Table 1.

**Table 1:** Descriptive data and correlations between sleep quality and quantity and lower-body neuromuscular performance characteristics.

Variable [unit]	x¯ ± SD	PSQI score		Hours of sleep	
		Pearson's r	*p*-value	Pearson's r	*p*-value
Concentric mean force [N]	1,809.8 ± 278.4	−0.830	<0.001*	−0.201	0.304
Relative concentric mean force [N/kg]	21.2 ± 2.6	−0.553	0.002*	−0.070	0.724
Concentric peak force [N]	2,327.0 ± 491.3	−0.679	<0.001*	−0.055	0.782
Relative concentric peak force [N/kg]	27.2 ± 4.9	−0.466	0.013*	0.063	0.749
Concentric mean power [W]	2,626.1 ± 453.8	−0.840	<0.001*	−0.249	0.200
Relative concentric mean power [W/kg]	30.9 ± 5.1	−0.514	0.005*	−0.135	0.493
Concentric peak power [W]	4,504.8 ± 681.5	−0.831	<0.001*	−0.346	0.071
Relative concentric peak power [W/kg]	53.1 ± 8.1	−0.424	0.025*	−0.209	0.287
Eccentric mean force [N]	2,242.5 ± 472.2	−0.610	<0.001*	−0.022	0.913
Relative eccentric mean force [N/kg]	26.3 ± 4.7	−0.375	0.049*	0.106	0.593
Eccentric peak force [N]	838.9 ± 104.3	−0.533	0.003*	−0.196	0.318
Relative eccentric peak force [N/kg]	9.8 ± 0.1	−0.052	0.794	−0.017	0.932
Eccentric mean power [W]	532.1 ± 112.5	−0.162	0.409	−0.117	0.553
Relative eccentric mean power [W/kg]	5.2 ± 1.1	0.199	0.311	0.311	0.107
Eccentric peak power [W]	1,765.5 ± 501.4	0.019	0.922	0.256	0.188
Relative eccentric peak power [W/kg]	20.9 ± 6.1	0.252	0.196	0.357	0.062
Vertical jump height [cm]	34.9 ± 6.0	−0.229	0.241	−0.269	0.166
RSI-modified [ratio]	0.5 ± 0.1	−0.567	0.002*	−0.056	0.777

RSI-modified, reactive strength index modified; PSQI, Pittsburg Sleep Quality Index.

* Statistically significant correlation (*p* < 0.05).

## 4 Discussion

The majority of the previous scientific literature has studied the impact of sleep on athletes’ aerobic, cognitive, and overall performance (11, 12, 14, 15, 26). However, to the best of our knowledge, this is one of the first investigations focused on examining the relationship between sleep quality and quantity, and lower-body neuromuscular performance characteristics within a cohort of semi-professional male basketball players. The findings of the present study revealed the presence of significant negative correlations between PSQI scores and various CVJ force-time metrics, indicating that better sleep quality is associated with greater eccentric and concentric force generation, concentric power production, and RSI-modified. However, no significant associations were detected between the sleep quality and absolute and relative eccentric mean and peak power, as well as vertical jump height. Lastly, when analyzing the relationship between sleep quantity (i.e., number of hours slept each night) and CVJ force-time metrics, no significant correlations have been observed.

Sleep quality is defined as “an individual's self-satisfaction with various aspects of the sleep such as efficiency, latency, duration, and wake after sleep onset” (27) and is of critical importance for optimal training adaptation and performance (28). Based on the findings obtained in the present investigation, a lower PSQI score is directly related to greater concentric force and power production. Also, sleep quality appears to have a greater impact on the concentric than eccentric phase of the CVJ. Specifically, while greater sleep quality is associated with greater eccentric force production, it does not seem to be associated with the movement velocity during the descending portion of the CVJ to the same extent, ultimately resulting in an absence of a significant relationship with eccentric mean and peak power (29). Despite being focused on examining acute fatigue-induced changes in athletes’ performance, a recently published investigation by Cabarkapa D et al. (30) found that in-season basketball practice of an approximate duration of two hours was capable of inducing a considerable decrease in multiple force-time metrics for only the concentric phase of the CVJ (i.e., concentric mean and peak force and power). Thus, it can be implied that the concentric phase might be more sensitive in detecting fatigue and recovery status than the eccentric phase of jumping motion. However, the eccentric phase should not be overlooked as it may provide practitioners with beneficial information when monitoring athletes longitudinally across a full competitive season span (31, 32).

While recently there has been a substantial debate if sleep quality is superior to sleep quantity, it is undeniable that they both are necessary components of regeneration and recovery (33). When focused solely on examining the quantity of sleep, Skein et al. (34) found that ∼30 h of sleep deprivation significantly decreased sprint performance. Also, Reyner & Horne (35) revealed that 5 h of sleep per night resulted in a 53% decrease in serving accuracy within a cohort of male and female tennis players when compared to a regular night's sleep (∼7 h) (36). However, contradictory findings have been obtained by Taheri & Arabameri (37), showing that short-term sleep deprivation did not cause any significant changes in the mean and peak power during the Wingate test. This is somewhat in line with our observations, where the amount of sleep that basketball players had each night throughout a one-month period did not have any significant association with their lower-body neuromuscular performance characteristics (e.g., concentric mean force, eccentric peak power). Thus, while further research on this topic is warranted, the aforementioned results suggest that focusing solely on sleep quantity may not be a reliable predictor of athletes’ overall sleep performance, implying that practitioners should consider incorporating both sleep quality and quantity in their analysis in order to get a more comprehensive understanding.

Although the findings of the present investigation offer valuable insight into the relationship between sleep and CVJ performance of semi-professional basketball players, this study is not without limitations. The sample of subjects who participated in the study was homogenous (i.e., male basketball players) and was examined at only a one-time point during the season (i.e., mid-season). Hence, future research should aim to examine if these findings remain applicable within different cohorts of athletes (e.g., female athletes, collegiate athletes), and sports (e.g., volleyball, handball), as well as if they stay consistent throughout the entire competitive season (e.g., longitudinal analysis). In addition, future research should examine if other factors such as dysregulations in circadian rhythms and disruptions in sleep habits due to frequent traveling and late-night game schedules impact athletes’ neuromuscular performance (3, 9).

In conclusion, sleep quality seems to have a greater impact on the concentric than the eccentric phase of the CVJ, while no significant relationship has been observed between sleep quantity and lower-body neuromuscular performance characteristics. Overall, these findings offer valuable insights into the importance of good sleep hygiene (e.g., efficiency, duration) in the athletic population, and can help practitioners design more effective training programs aimed at mitigating injury risks associated with accumulated fatigue and inadequate recovery.

## Data Availability

The raw data supporting the conclusions of this article will be made available by the authors, without undue reservation.
